# The utility of the abdominal series in the emergency setting: a retrospective review

**DOI:** 10.1186/s12245-023-00580-3

**Published:** 2024-01-04

**Authors:** Sarah Hogan, Joshua Ward, Eric Sala

**Affiliations:** Memorial University of Newfoundland, St. John’s, NL Canada

**Keywords:** Abdominal radiology, Emergency radiology, Emergency medicine, Resource utilization

## Abstract

**Purpose:**

The abdominal series (AXR) remains a frequently ordered test in the emergency department (ED), despite existing literature questioning its utility. The aim of this study was to characterize the use of the AXR in the ED by quantifying how often it is ordered and the frequency of subsequent imaging. Additionally, a time estimate in ED associated with the AXR was quantified. We hypothesized that there would be a low clinical utility of the AXR, and long associated time period spent in the ED.

**Methods:**

A retrospective audit of AXRs performed in the ED from January to December 2019 was performed. The local picture archiving and communication system (PACS) and electronic medical record were used to collect the variables.

**Results:**

Of 701 AXRs, 438 (62.4%) were reported normal, and 263 (37.6%) were abnormal. A Chi Squared test showed that the two variables (abdominal series result and follow up imaging completion) were significantly related, with *p* < 0.001. However, the effect size was small (Nagelkerke R square = 0.022). The average time spent in the ED for these patients was 7.27 h, and the average time between the AXR being ordered and interpreted was 1.31 h.

**Conclusion:**

The majority of AXRs were reported as normal. Our results showed that AXR had a statistically significant, but low clinically significant predictive ability on subsequent imaging ordering. This supports our hypothesis that the AXR is of low clinical utility with respect to the rate of ordering follow up imaging. The AXR also translated to a quantifiable time interval during the patient’s stay in ED. Minimizing overuse of the AXR may result in a decrease in patient duration in the ED.

## Introduction

Acute abdominal pain remains a common presentation to the emergency department [1–4] and the abdominal series is a commonly ordered test, with up to 52% of patients presenting with acute abdominal pain undergoing an abdominal series [5, 6]. Diagnostic imaging in general is steadily increasing in utilization in the emergency department [7, 8]. Despite its persistent use, the utility of the abdominal series has been questioned in multiple prior studies, [9, 10]. Prior studies date back to 1982 at which point Eisenberg et al. determined that the diagnostic rate of the plain abdominal series was low, at only 10% [11]. The nondiagnostic rate of the abdominal series has been estimated to be as high as 68% [11, 12]. Similarly, the abdominal series has been shown to yield a high proportion of non-specific findings, up to 46% [5, 10]. More recently, the prevalence of clinically significant findings identified on the abdominal series has been estimated to be as low as 12% [9], and is only estimated to help with diagnosis in 2–8% of cases [10]. Prior researchers have suggested that the utility of the abdominal series is so low, that the emergency department staff should forgo its use altogether, and seek more definitive imaging earlier in presentation [10]. Accordingly, recent data has shown that the use of abdominal CT is steadily increasing [13, 14], and results in higher diagnostic accuracy [15, 16].

A high proportion of patients who undergo an abdominal series proceed to have further imaging, as high as 57% [5, 9, 10]. Patients are more likely to proceed to further imaging if they have an abnormal abdominal series [9, 10]. In fact, radiologists recommend further imaging for the abdominal series in as many as 10% of cases [17].

Even in the earlier days of radiology, prior to easy access to computed tomography, the indiscriminate use of the abdominal series was questioned, and recommended to only be used in patients with high clinical suspicion of bowel obstruction, renal calculi, trauma, or ischemia [11]. The abdominal series has a particularly low sensitivity for pathologies including pancreatitis, appendicitis, pyelonephritis, and diverticulitis [12]. The sensitivity of the abdominal series has been established to be highest for the evaluation of bowel obstruction and foreign body ingestion [12, 18]. A large recent review article indicated that the only current indications for a plain abdominal series are acute abdominal pain in the setting of suspected obstruction, or in the case of suspected foreign body [19], grossly shortening a prior list of indications provided by the Royal College of Radiologists [20]. More recent data has indicated that there is essentially no role for the abdominal series in the workup of the acute patient [6, 21]. Despite this previous work, there remains persistent overuse of the abdominal series in the emergency department [22–25].

Computed tomography has been shown to be superior for the evaluation for abdominal pain [16, 26], and accordingly, has become steadily more commonly ordered in recent years [27, 28]. Current guidelines exist which outline the appropriate use of abdominal radiography in the clinical setting. The Canadian Association of Radiologists (CAR) has published guidelines regarding the appropriate imaging modality for a variety of gastrointestinal pathologies. These guidelines state that the abdominal series is indicated for small bowel obstruction, inflammatory bowel disease, the acute surgical abdomen, and acute abdominal pain as a second line of investigation if CT is not available. CT is the superior imaging test for these pathologies [29]. Similarly, the ACR (American College of Radiology) states that a CT of the abdomen and pelvis is “usually appropriate” for acute non localized abdominal pain, and plain abdominal radiography is classified as “may be appropriate” secondary to its low overall sensitivity for etiologies other than foreign bodies and bowel obstruction [30].

Obtaining these images are not benign and come with risk to the patients and even others working in the department. The risk of exposing individuals to radiation must be weighed against the potential benefit to the patient. The abdominal series generally includes supine and erect abdominal radiographs, as well as an erect chest x-ray [31, 32]. In recent years, the chest radiograph has been excluded from the abdominal series at some sites in an attempt to reduce radiation dose [33]. Each abdominal radiograph has a radiation dose of 0.7 mSv, for a total of 1.4 mSv for both supine and erect [34]. This is significantly higher than the radiation dose of 0.02 mSv for the chest radiograph, resulting in a total of 1.42 mSv for the abdominal series [32, 34]. For comparison, a low dose CT (LDCT) of the abdomen is about 2–3 mSv [35]. Furthermore, beyond the small increase in radiation using the LDCT, Nguyen et al. found the LDCT had a better yield diagnostically, thus resulting in a lower number of follow up imaging [35]. This has been corroborated by multiple other studies [6, 36, 37],

While multiple studies have outlined the low diagnostic utility of the abdominal series, there is more limited literature regarding the impact the use of abdominal radiography has on patient wait times in the emergency room and delays in obtaining more definitive imaging. The current literature has established that a large portion of patients who undergo abdominal radiography go on to have further imaging such as CT or ultrasound [5, 9, 10]. Therefore we hypothesize that the use of the abdominal series will translate to a quantifiable delay in diagnosis and increase in time spent in the emergency room. We aimed to characterize current use of the abdominal series in our regional emergency department with this project, and to attempt to quantify the time spent in the emergency department related to the abdominal series. We hypothesized that we would demonstrate a low utility of the abdominal series in the emergency department, similar to prior studies, which would lend support to the use of more definitive imaging modalities such as CT or ultrasound earlier in the patient’s presentation.

## Methods

This study was designed as a one-year retrospective audit. Approval from the local Health Research Ethics Board was obtained prior to initiation of data collection.

Every consecutive patient who underwent an abdominal series in the emergency room at the Health Sciences Centre, the regional tertiary care center, was included, between January 2019 and December 2019. Exclusion criteria included patients under 18 years of age, radiographs used for device placements, any abdominal series that occurred as follow up to other imaging, patients that were direct admission to other services, and unclear time stamps on acquired images. This timeframe was chosen because it predated the COVID-19 pandemic and was recent enough that the results would reflect current emergency room patterns. Based on prior literature, we aimed for a sample size of *n* = 1000. With an average of 100 abdominal series performed per month at the regional emergency department, we anticipated that data saturation would occur within one calendar year.

Data collection was performed using the local Picture Archiving and Communication System (PACS), and the local Electronic Medical Record (EMR). The collected variables were as follows: date and time of emergency room check-in, date and time of emergency room discharge or admission to service, age, sex, time of abdominal series order, time of abdominal series image acquisition, time of preliminary read by emergency room physician, result of preliminary read by emergency room physician (normal or abnormal), result of final read by radiologist, pathology reported on abdominal series, if follow up imaging was performed within the same visit, time of follow up imaging order, time of follow up imaging acquisition, result of follow up imaging (normal or abnormal), pathology on follow up imaging.

The “end point” of the patient’s stay in the emergency department was determined as either the time of discharge or time of admission to a hospital service. The “time to decision” on the results of the abdominal series was estimated by the time stamp of the preliminary read note entered onto the PACS by the emergency physician. If this note was absent, but the radiologist’s final report was published within the hour, this timeframe was estimated to be one hour. We noted that when the emergency physician’s initial read was present, it was posted on average within one hour of the study being performed. Therefore, we assumed that if the radiologist’s report was posted within one hour, and the emergency initial read was absent, then the emergency physician likely read the radiology report at the time of interpretation, thereby negating the need to document their own initial read.

A “normal” abdominal series was defined as any radiograph that was reported as normal by the emergency physician on the PACS preliminary read, or which was reported as normal by the reporting radiologist if the report was published within an hour. Similarly, an “abnormal” abdominal series was defined as any radiograph that was reported as abnormal by the emergency physician on the PACS preliminary read, or which was reported as abnormal by the reporting radiologist if the report was published within an hour.

Statistical analysis was performed using IBM SPSS Software Version 21 (Armonk, NY). Initially, a Chi Squared test was performed comparing the result of the abdominal series with the completion of follow up imaging. A binary logistic regression was also performed, comparing the same variables.

## Results

A total of 1096 abdominal series were collected in the data set. There were 617 female patients, and 477 male patients. The average age was 54.7 years, spanning from 18 to 99 years. Overall, 297 (27%) patients went on to have follow up imaging in the form of CT or ultrasound. The majority of follow up imaging was in the form of CT (237 cases, 79.8%).

The average time span between patient presentation to the emergency room and discharge/admission was 7.2 h. The average time span between the patient presentation to the emergency room and the abdominal series being ordered was 2.9 h. The average time span between the abdominal series being ordered by the emergency physician and it being completed was 0.5 h (Table 1).

**Table 1:** Average durations for key points in duration of ED stay

Total duration in ER (triage time to discharge/admission)	7.2 h
Average time between triage time and AXR ordered	2.9 h
Average time between AXR ordered and completed	0.5 h
Average time between AXR completed and being interpreted (either ER preliminary note, or if radiologist report published within one hour)	1.31 h

In order to evaluate the patterns of subsequent imaging ordering based on the initial interpretation of the abdominal series, a subset of data was created. The estimate of the time and content of first interpretation was made based on the presence of the emergency physician's preliminary read note documented on the PACS system. If there was no emergency physician preliminary read, but the radiologist’s final report was published within one hour, we assumed that the radiologist’s report was read by the emergency room physician, and they therefore opted not to include the preliminary read. A total of 701 abdominal series had an associated emergency physician preliminary read note, or alternatively, a radiologist report published within one hour of the study being acquired.

**Fig. 1:**
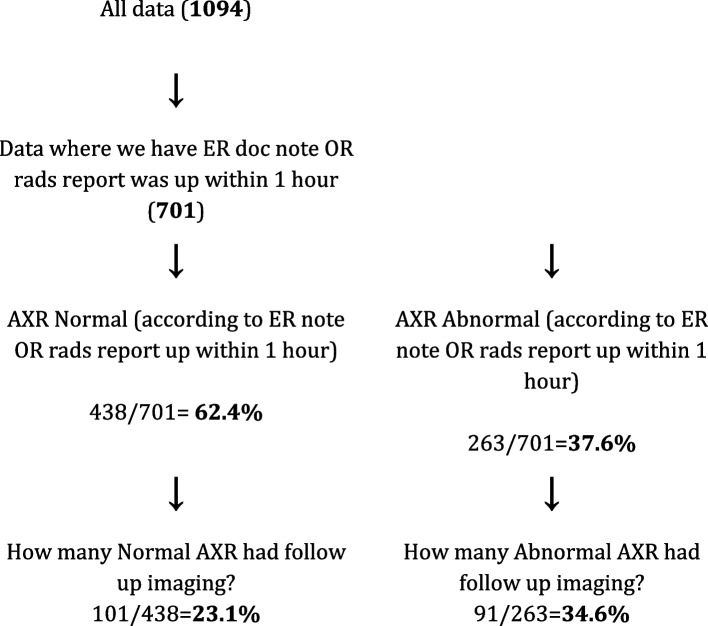
Frequency of follow-up imaging for radiographs reported as normal and abnormal

**Fig. 2:**
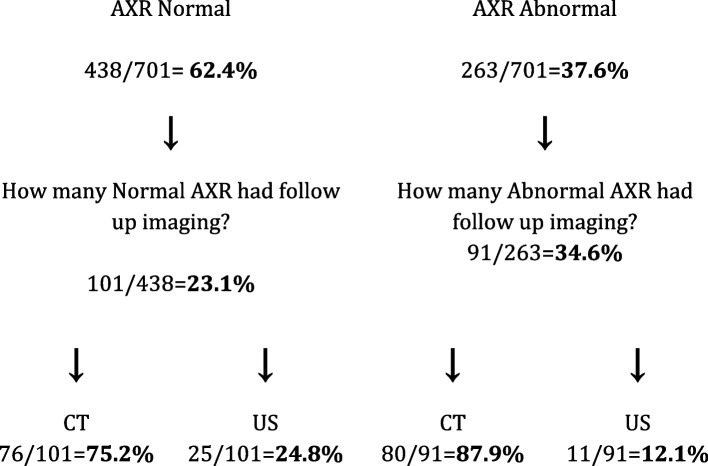
Type of follow up imaging for AXR reported as normal and abnormal for AXR with ER preliminary note or radiologist report published within one hour

A total of 438 (62.4%) abdominal series were reported as normal by either the emergency physician preliminary read or radiologist report if it was published within one hour. Of this group, 101 (23.1%) went on to have follow up imaging (Fig. 1). Of the follow up imaging, there were 76 CTs, and 25 ultrasounds (Fig. 2).

A total of 263 (37.6%) abdominal series were reported as abnormal by either the emergency physician preliminary read or radiologist report if it was published within one hour. Of this group, 91 (34.6%) went on to have follow up imaging (Fig. 1). Of the follow up imaging, there were 80 CTs and 11 ultrasounds (Fig. 2).

A Chi Square test was completed to compare the abdominal series result and follow up imaging completion. The Chi square test showed that these two variables were significantly related (*p* < 0.001). However, the effect size was small, with a Nagelkerke R square = 0.022. Subsequently, a binary logistic regression was performed on the same data. The binary logistic regression demonstrated that the result of the abdominal series significantly predicted the frequency of subsequent imaging (*p* < 0.01), however, the effect size was still small at *R* = 0.022.

The average time span between the abdominal series being ordered at first interpreted by the emergency room (either as timestamp of the preliminary read or estimated to be 1 h if the radiologist report was published within 1 h) was 1.31 h, ranging from 0.17 to 8.4 h (Table 1).

There were 574 abdominal series with a documented preliminary read by the emergency room physician. The most common preliminary read by the emergency room physician was “Nil acute” (381, 66.4%), followed by “Nonspecific bowel gas pattern” (83, 14.5%), “stool” (i.e. any comment on retained stool or constipation) (52, 9.1%), and “SBO/ileus” (28, 4.9%). The remaining recorded pathology included nephrolithiasis, cholelithiasis, foreign body, and other entries as outlined in Table 2.

**Table 2:** Pathology on AXR as reported by ER physician

Nil acute	381	66.4%
Nonspecific bowel gas pattern	83	14.5%
Small bowel obstruction/Ileus	28	4.9%
Stool	52	9.1%
“Pathology seen”	12	2.1%
Nephrolithiasis	5	0.87%
Opacity	2	0.35%
Mass	1	0.17%
Diverticulitis	1	0.17%
Cholelithiasis	1	0.17%
Gastric distension	1	0.17%
Hernia	1	0.17%
Bowel wall thickening	1	0.17%
Volvulus	1	0.17%
Bezoar	1	0.17%
Stent	1	0.17%
Foreign body	1	0.17%
Ground glass	1	0.17%

There were 127 abdominal series with a final radiologist report published within one hour of the scan being taken in the absence of an emergency physician preliminary read. The most common report by the radiologist if the report was published within one hour of the abdominal series being ordered was “nil acute” (67, 52.8%), followed by “stool” (25, 19.7%), “nonspecific bowel gas pattern” (20, 15.7%), and “SBO/ileus” (9, 7.1%). Other reported entries are outlined in Table 3.

**Table 3:** Pathology on AXR as reported by Radiologist if within one hour

Nil acute	67	52.8%
Nonspecific bowel gas pattern	20	15.7%
Small bowel obstruction/Ileus	28	22.0%
Stool	25	19.7%
Foreign body	2	1.6%
Gastric outlet obstruction	1	0.79%

Overall, there were 172 cases with a reported abnormal AXR which did not go on to have follow up imaging. The most common pathologies identified on the abdominal series by either the preliminary ER physician read or radiologist if reported within one hour which did not go on to have follow up imaging were “Stool” (any comment on fecal loading, retained stool, constipation), (71, 41.3%), “Nonspecific bowel gas pattern” (including phrases such as “nonspecific air fluid levels”, “gas seen throughout the bowel”, “nonspecific air fluid levels”) (59, 34.3%), and “SBO/Ileus” (19, 11.0%). Other less common descriptors are outlined in Table 4. There were no reports of free air.

**Table 4:** Pathology on AXR if no follow up imaging

SBO/Ileus	19	11.0%
Stool	71	41.3%
Nonspecific bowel gas pattern	59	34.3%
Nonspecific comments such as “Unchanged”, “Pathology seen”, etc	8	4.7%
Foreign Body	3	1.7%
Gastric distension	2	1.1%
Mass or opacity	2	1.1%
Extra abdominal comments such as “Consolidation”, “osteoarthritis”	2	1.1%
Nephrolithiasis	2	1.1%
Cholelithiasis	1	0.6%
Hernia	1	0.6%
Volvulus	1	0.6%
Diverticulitis	1	0.6%

Overall, there were 91 cases with a reported abnormal AXR which did go on to have follow up imaging. The most common pathologies identified on the abdominal series by either the preliminary physician read or radiologist if reported within one hour which did go on to have follow up imaging were “Nonspecific bowel gas pattern” (including phrases such as “nonspecific air fluid levels”, “gas seen throughout the bowel”, “nonspecific air fluid levels” (43, 47.3%) and SBO/Ileus (18,19.8%). Stool was only commented on in 7 (7.7%) cases. Other descriptors are outlined in Table 5.

**Table 5:** Pathology on AXR if yes follow up imaging

Nonspecific bowel gas pattern	43	47.3%
SBO/Ileus	18	19.8%
Nonspecific comment such as “Unchanged”, “patient note”, “pathology seen”	12	13.2%
Stool	7	7.7%
Nephrolithiasis	5	5.5%
Thick wall	1	1.1%
Ground glass	1	1.1%
bezoar	1	1.1%
Opacity in pelvis	1	1.1%
Stents	1	1.1%
Gastric Outlet Obstruction	1	1.1%

## Discussion

This data demonstrated a significant predictive effect of the abdominal series on subsequent imaging (*p* < 0.01) according to a binary logistic regression, and a significant relationship between the abdominal series and subsequent imaging according to a chi square test (*p* < 0.01). However, the effect size, R, was small for both tests, at only 0.022, meaning that the abdominal series only accounted for 2.2% of the variance regarding follow up imaging. This means that there was a statistically significant, but low clinically significant predictive ability on subsequent imaging taking place. This supports our hypothesis that the abdominal series is of low clinical utility with respect to the rate of follow up imaging.

Of the patients who went on to have follow up imaging after an abnormal abdominal series, the most common pathology reported on the AXR was “Nonspecific bowel gas pattern” and SBO/ileus. Of the patients who did not go on to have follow up imaging, the most common pathology reported on the AXR was “Stool”, “Nonspecific bowel gas pattern”, and “SBO/Ileus”. The AXR therefore was generally of low clinical utility for determining the use of follow-up imaging, although may have been helpful in the case of fecal loading.

This data supports the existing literature, which states that the clinical utility of the abdominal series is low [9, 21]. As well, an abdominal radiograph reported as normal did not preclude a patient from receiving further diagnostic imaging, as 23.1% of these patients still went on to have a CT or US in the same visit.

The majority of initial reads of the abdominal series by either the ER physician or radiologist were reported as normal (62.4%). Even of the abnormal radiographs, the majority were reported as “nonspecific gas pattern” (14.6% and 15.7% for ER physicians and radiologists respectively), which has been discouraged as a radiological descriptor in prior research due to its lack of clarity and clinical guidance [38, 39].

One of the limited remaining indications for the plain abdominal series according to some sources is suspected small bowel obstruction or ileus, which was only identified in 4.9% of radiographs reported by ER physicians, and 7.1% of radiographs reported by radiologists. This is in line with current literature, which reports relevant findings from the abdominal radiograph are only present in 4–12% of cases [40, 41].

The estimated time period associated with the abdominal series from the time point at which it was ordered, and the time at which it was estimated to be interpreted by the emergency physician was 1.31 h. While the emergency physician is likely occupied with other tasks during this time, and this delay may not be avoidable, it is reasonable to assume that ordering and interpreting the abdominal series does add some time to the emergency visit. This corresponds with existing data, which demonstrates that diagnostic imaging adds up to 1 h to the patients expected length of stay [42–45]. With an average total time in the emergency department of 7.27 h, this corresponds with 18.0% of the total stay in the emergency department. This is an updated attempt to quantify the time associated with ordering an abdominal series in the emergency department. In the setting of rapidly increasing wait times in the emergency department [44, 46] and increased morbidity associated with longer wait times, the abdominal series may be helpful as a target to reduce less useful and time consuming investigations. This would conceivably allow patients to move through the emergency department more quickly. In addition to increasing the length of stay in the ER, there is increased radiation exposure to patients who receive both the abdominal series and LDCT when compared to only the LDCT, 3.42–4.42 mSv versus 2-3 mSv, respectively [32, 34, 35]. The occurrence of advanced medical imaging at the time of emergency department visit has been shown to reduce re-visit rates to the ED and reduce further imaging rates [47]. Our data supports the current literature outlining the poor utility of the abdominal series, and we recommend the use of more definitive imaging in the emergency department according to current guidelines, in lieu of the abdominal series.

There were several limitations of this study. Not all of the collected abdominal series had an emergency physician preliminary read entered on PACS (64.1%), which limited the ability to estimate the timeline in the emergency department associated with the abdominal series. Unfortunately, due to the limited nature of the local EMR, there was no other way to determine the emergency physician’s interpretation of the abdominal series.

If the abdominal series was reported by a radiologist with a published report within one hour, we assumed that the emergency physician read that official report, and therefore did not need to enter a preliminary read. This assumption is a limitation and may not have always reflected the true sequence of events in the emergency room. However, it was determined to be the most accurate assumption we could make within the limits of a retrospectively designed study and allowed us to include the largest possible sample size.

If there was no preliminary read, and no radiologist report within one hour, the abdominal series was excluded from the data analysis. Throughout our data collection, we noted that the time stamps on the abdominal films were obviously incorrect, for example occurring before the abdominal series was entered by the emergency department. We were therefore limited by the time calibration of the x-ray machines and had to assume that they were correctly calibrated for the remainder of the data points. Additionally, the reports published by the radiologists and notes reported by the emergency physicians were provided by a variety of individuals employed at our institution. While all physicians at our site have similar background in terms of their Canadian medical training, and in the case of radiologists, Royal College certification, this would certainly create an unavoidable degree of inter-rater variability. Finally, we only recorded any subsequent imaging that occurred within the same emergency department visit. Any patients that went home and returned to the emergency department the next morning for follow up imaging were not included. These patients who receive next day follow up imaging, must then return to the emergency department, once again, for a separate visit to obtain their reports, thus contributing to the ER patient burden.

## Conclusion

 Our data adds support to the current literature regarding the low clinical utility of the abdominal series in the emergency department. In the setting of advancing low dose CT techniques and known improved outcomes in patients with more definitive advanced medical imaging in the emergency department when indicated, the abdominal series should be avoided in the acute setting. The abdominal series also corresponds with a quantifiable time frame in the emergency department, and in the setting of increasing wait times, proceeding to more definitive imaging initially may reduce the overall length of stay in the emergency department for these patients.

## Data Availability

Data sets can be requested for review by contacting the corresponding author at seh663@mun.ca.
